# Christianity, Social and Joyous

**Published:** 1864-08

**Authors:** 


### CHRISTIANITY, SOCIAL AND JOYOUS.

It is true that Christians are in and not of the world, but they are not therefore to become ascetics and remain gloomily isolated from all society, denied to the comforts and pleasures of earth. There is no "must needs go out of the world" imposed upon the friends of Jesus, but an imperative, "Go ye into all the world," and by a pure and elevated social and religious intercourse, "preach the gospel to every creature." Thus, by the manifestation of the truth in our lives, we will commend ourselves and our religion to every man's conscience.

There are two errors just here. *The first:* that religion requires and produces in its subject an austere and harsh countenance on which no smile ever plays, the compressed lip, contracted brow, unrelaxed muscles and ever present tear-drop; in fine, a "touch-me-not" expression and action towards every person who has not a sad and disfigured face consonant with our own; *the second*: that sorrow, tears, fear, humility and grief, belong only to the unconverted and not at all to the renewed soul. It makes conversion a synonym for assured hope and unalloyed joy and gladness, and requires the foul-mouthed blasphemer of yesterday and convert of to-day to be recognized as a convert, to possess the high-emotioned experience of "assurance of God's love, peace of conscience and joy in the Holy Ghost, which pertains to the man who has grown hoary in the service of the Redeemer, and through a varied experience of sunshine and shade has attained the high pinnacle, "ready to be offered, and the time of departure at hand" Between these two opposite extremes of melancholy and fanatical joy, there is a medium at which every one should aim, and to the attainment of which true godliness will gradually but surely prompt and guide our effort. I have said, gradually, "for every soul carries within itself treasures of sorrow," from whose fountains come only anguish and bitterness until a mightier than Moses cast in the tree whose leaves are life-giving, or the healing salt. These instantaneously find their place in the secret sources, and mingling with the waters, affect sensibly the fountain; but it is a work of time to so change that the stream shall be wholly pure. This will still be the characteristic of the renewed heart; its sorrow will not be all sorrow, its joy will not be without tears and mourning. Hear the royal Psalmist: "Light is sown for the righteous and gladness for the upright," *but*, "Rejoice with trembling;" and the royal Preacher: "It is better to go to the house of mourning, than to the house of feasting;" yet, "pleasant words are as a honey comb, sweet to the soul, and health to the bones." The Saviour combines these thoughts in that wonderful and precious benediction uttered from Mount Gerizim: "Blessed are they that mourn, for they shall be comforted."

The renovated soul has not lost any of the many fruitful sources of mental and physical suffering, but has an additional one in that *sorrow for sin* which interpenetrates the soul in every emotion and action, giving it all that humble sadness and serious thoughtfulness which, like a veil, overspreads and colors the joys it cannot conceal. But contemporaneous with this, there are new principles and opposite forces imparted, which by attraction and propulsion, elevate the soul and fill it with joy, alleviating every pain and sorrow. Through their transforming power, the sorest chastisements become a precious oil, and all afflictions are counted light, because they "work out an eternal weight of glory." Here social sympathy too comes in, bearing relief and consolation; for sorrow shared is lightened, while joys divided are doubled. There is a giving that does not diminish, a withholding that does not increase; a giving that is more blessed than to receive—the Bible announces the fact, the true Christian life solves the problem and proves it. Christianity then does not forbid society, but demands it, elevates it, purifies it by restricting all that is low and trifling in its character, all that wounds the sensitive spirit and the tender conscience, by furnishing whatever promotes and favors it. Religion is appropriate—is an element of joy and gladness around the "Fireside," at the watering place, in the car and steamboat, as well as in the closet and in the house of God.

J. C. K. M.

---

WHATEVER you have to do, do promptly, cheerfully, and well.

